# LDTs in Flux: Will Evolving Regulations Enhance or Complicate Laboratory Practices?

**DOI:** 10.21627/ggcs2a65

**Published:** 2026-07-06

**Authors:** Alejandro R. Molinelli, Dennis J. Dietzen, Jonathan R. Genzen, Xander M. R. van Wijk, Maria Alice V. Willrich, Melissa M. Budelier

**Affiliations:** Department of Pharmacy and Pharmaceutical Sciences, St. Jude Children’s Research Hospital, Memphis, TN, United States; Phoenix Children’s Hospital, University of Arizona College of Medicine, Phoenix, AZ, United States; Department of Pathology, University of Utah, Salt Lake City, UT, United States; ARUP Laboratories, Salt Lake City, UT, United States; Department of Laboratory Medicine, University of California San Francisco, San Francisco, CA, United States; Department of Laboratory Medicine and Pathology, Mayo Clinic, Rochester, MN, United States; TriCore Reference Laboratories, Albuquerque, NM, United States; Department of Pathology, University of New Mexico, Albuquerque, NM, United States

**Keywords:** Laboratory developed test, FDA, regulatory, quality, CLIA

## Abstract

Clinical labs help diagnose and manage disease by testing blood and other specimens. They operate under federal rules known as Clinical Laboratory Improvement Amendments (CLIA). Most tests are FDA cleared/approved, but some are laboratory-developed tests (LDTs) created in individual labs, typically to meet unmet needs. LDTs have long existed in a complicated regulatory space. In 2024, the FDA issued a rule to regulate LDTs like devices, prompting concerns about cost, innovation, and access. A federal court struck the rule down in 2025, finding LDTs are services, not devices. The FDA subsequently rescinded its rule. In this article, experts argue that LDTs are essential and already held to strong standards and that future reforms should strengthen CLIA while protecting access and innovation.

Clinical laboratories perform analyses of human biological specimens to aid in the diagnosis, management, and treatment of patients. In the United States, the operation of clinical laboratories and the qualifications of their personnel are regulated under the Clinical Laboratory Improvement Amendments (CLIA) of 1988, which are administered under the oversight of the Centers for Medicare & Medicaid Services (CMS).

Tests performed in clinical laboratories generally fall into one of two categories: in vitro diagnostic (IVD) devices or laboratory-developed tests (LDTs). IVDs are commercially manufactured assays that, in the United States, are subject to pre-market and post-market regulatory controls under the oversight of the US Food and Drug Administration (FDA) to ensure their safety and effectiveness. IVDs comprise the majority of tests performed in clinical laboratories. In contrast, LDTs are developed and used within a single laboratory and are not cleared or approved by the FDA. LDTs are typically designed to address unmet clinical needs. For example, mass spectrometry–based LDTs are essential for providing accurate results in the disciplines of toxicology, the detection of markers of inborn errors of metabolism, and proteomics.

The regulation of LDTs is complex and evolving [1]. Since the enactment of the Medical Device Amendments of 1976, the FDA has maintained regulatory authority over IVDs. The FDA has considered LDTs to be a subset of IVDs that fall within its regulatory purview; however, it has historically exercised *enforcement discretion* by choosing not to actively regulate them. The FDA defines an LDT as an “in vitro diagnostic test that is manufactured and used within a single laboratory” [2]. In October 2014, the agency published guidance documents outlining a framework for increased regulatory oversight of LDTs. However, these guidance documents were withdrawn in November 2016. Between 2018 and 2022, multiple versions of the Verifying Accurate Leading-Edge IVCT (In Vitro Clinical Tests) Development Act (VALID Act) were introduced in Congress, each proposing a legislative framework to bring LDTs under FDA oversight [2–4]. All attempts to pass the VALID Act failed to achieve consensus in Congress. The most recent effort occurred in December 2022, when the bill was excluded from an end-of-year omnibus spending package. In May 2024, without congressional action, the FDA issued a final rule that would have phased out enforcement discretion for LDTs and caused these tests to fall under the same enforcement framework as commercially marketed IVDs [5–6]. The final rule included provisions encompassing medical device reporting, registration and listing requirements, selected elements of the quality system regulations, and a risk-based phase-in of premarket review that would have imposed significant financial and operational burdens on clinical laboratories. Ultimately, this rule would likely have led to the curtailment of important tests for patients. The American Clinical Laboratory Association and the Association for Molecular Pathology sued the FDA, and on March 31, 2025, the US District Court for the Eastern District of Texas ruled in favor of the plaintiffs, concluding that LDTs are medical test services and that regulating LDTs as devices exceeds the FDA’s regulatory authority. The court vacated the final rule in its entirety [7]. In August 2025, the FDA issued a notice to the Office of Information and Regulatory Affairs signaling its intent to rescind the rule.

Given the history of LDT regulation, this topic will likely remain mired in uncertainty. Following these recent developments, and as a follow-up to the 2023 *JMSACL* special issue on laboratory-developed tests, we invited several experts from academic and non-profit reference laboratories to share their perspectives on the current regulatory landscape and the potential future of LDT oversight (Figure 1).

**Figure 1.**
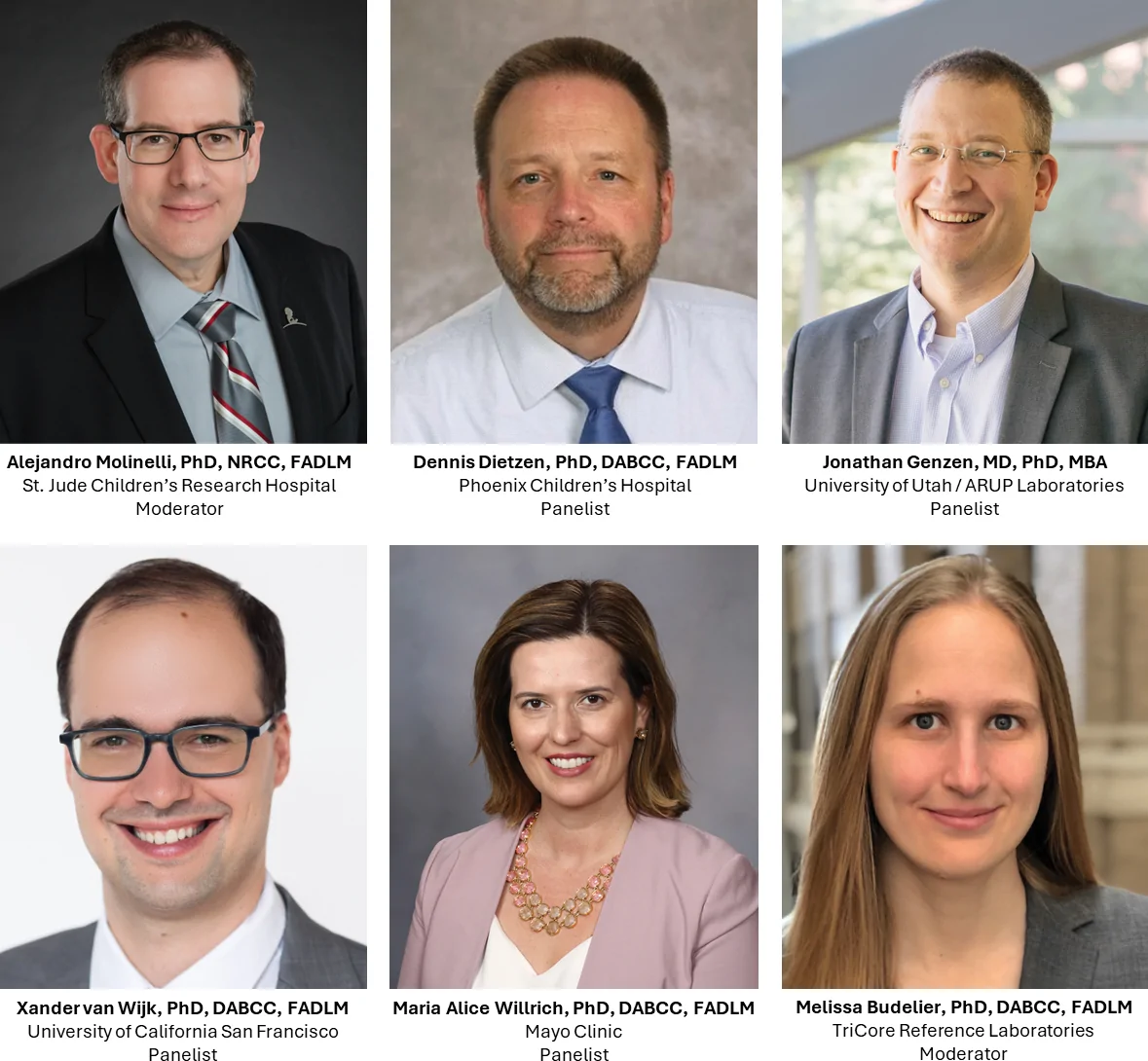
Panelists and moderators.

**What is the scope of laboratory developed tests performed at your laboratory and the potential impact the FDA final rule would have had? Please describe the impact to your organization of the final rule being struck down.**

**Dietzen:** Laboratories like mine that serve pediatric clinicians and patients were especially vulnerable under the final rule. The diagnostic marketplace has been driven to high volume, largely adult environments, rather than pediatric environments, where diseases are rare and sample volumes are small. For this reason, pediatric practitioners frequently need to build their own tools (i.e., LDTs). The measurement systems we build include LDTs for pediatric cancers, inborn errors of metabolism, therapeutic drugs, illicit drugs, and constitutional genetic disorders. The analytic tools we use include flow cytometers, mass spectrometers, automated chemistry analyzers, tissue stainers, and gene sequencers. Pediatric use of LDTs is even more expansive than it appears. Many of the FDA-approved methods we use were not tested in children, so the use of almost any reagent system in pediatrics could, in one sense, be considered laboratory developed. As John F. Kennedy said about going to the moon, we pursue pediatric lab medicine “not because it is easy, but because it is hard.” The LDT final rule would have made this already challenging work even harder.

**Genzen:** ARUP Laboratories is a nonprofit enterprise of the University of Utah and its Department of Pathology. Along with being a national clinical reference laboratory, we also operate our health system’s inpatient and outpatient laboratories. As such, we have LDTs that cover the entire spectrum of diagnostics and laboratory settings, from esoteric testing for rare disorders all the way to routine body fluid tests that are considered LDTs because the source types have not previously been validated by an in vitro diagnostic (IVD) manufacturer. We have been very open in our advocacy efforts describing the importance of LDTs, not just in our laboratory but also across the broader clinical laboratory community. LDTs are particularly useful for rare disorders and situations where there is not a financial market for an IVD manufacturer to justify producing kits or validating less-common specimen types.

As a national reference laboratory, our customers depend on us to develop and maintain LDTs. The final rule being struck down enables us to dedicate additional resources toward innovation and ongoing patient care by supporting the depth and high quality of our existing and future menu of laboratory tests, including LDTs.

**Van Wijk:** UCSF Health is an academic medical center with multiple hospitals and clinics across the San Francisco Bay Area. The Clinical Chemistry section of UCSF Health Clinical Laboratories performs mass spectrometry–based LDTs for purposes such as therapeutic drug monitoring (TDM) and androgen/estrogen testing. One area of impact is busulfan testing, which we perform for both the adult and pediatric populations. Personalized dosing with TDM has been shown to decrease severe toxicities, graft rejection rates, and relapse rates. Busulfan concentration measurements require a fast turnaround because they are the basis for the calculation of the next busulfan dose. To my knowledge, there is currently no FDA-cleared or FDA-approved test available for measurement of busulfan.

The FDA final rule would have prevented us from developing additional tests and updating existing ones. Now that the final rule has been struck down, we are able to move forward with projects such as incorporating automation into sample processing for LDTs such as testosterone testing.

**Willrich:** We perform LDTs in different areas of the clinical laboratories I oversee, and overall at my institution, 50% of tests on our menu are classified as LDTs. I have developed tests for monoclonal antibody therapies (t-mabs) such as infliximab, adalimumab, vedolizumab, ustekinumab, and others used to manage inflammatory bowel diseases and rheumatological conditions. We have been pioneers in bringing several of these tests into the clinical laboratory.

LDTs are also crucial for complement testing. We rely on LDTs for analysis of specific complement activation fragments, to assess the activity of other complement pathways, and to detect autoantibodies to complement components. These tests are all low volume but critically important for diagnosing rare disorders and guiding high-stakes treatment decisions.

Another impactful LDT in my laboratory is MASS-FIX, a MALDI-TOF method for detecting monoclonal proteins, originally developed at Mayo Clinic around 2014 and clinically implemented in 2018 as an LDT. We have thus far analyzed more than 500,000 cases with this technology and have identified new risk factors for disease progression, such as light chain glycosylation. We have added approximately 10 fold sensitivity compared with serum immunofixation. The added sensitivity may reduce the need for bone marrow monitoring for minimal residual disease and has created many ripples in the monoclonal gammopathy field.

The FDA’s final rule would have substantially increased the regulatory burden for these and many other tests, potentially limiting our ability to innovate and respond rapidly to patient needs. The rule being struck down preserves flexibility to continue developing and offering these high-value, clinically impactful LDTs without imposing additional costs and barriers that could have undermined access to care.

***What do you think potential/future legislative action could look like? Is there anything we should be doing to prepare ourselves for this?***

**Dietzen:** In my mind, there are three possibilities that may play out in the near-to-intermediate term. In order of likelihood (1 being least likely), they are:

1) A deliberate and thoughtful revision of CLIA regulations to bolster the real and perceived deficits in the LDT validation process. Some actors, bad ones mostly, have taken refuge behind the LDT process to minimize or escape regulatory scrutiny. This is rare, but it should not happen. This CLIA-centric approach, supported by the Association for Diagnostics & Laboratory Medicine (ADLM), the American College of Medical Genetics and Genomics (ACMG), and the Association for Molecular Pathology (AMP), among others, relies on the influence of the Clinical Laboratory Advisory Committee (CLIAC) to develop and promulgate change. With the recent disbanding of the CLIAC, I see this as a remote possibility.
2) There could be a replay of the VALID Act or a similar legislative remedy [4]. Many of the original sponsors of this bill may try to take another ride at passage.
3) The most likely course of action in the near future is nothing. The current chaos and anti-regulatory fever in Congress are unlikely to change soon. Regulatory agencies are being handicapped or outright gutted. Laboratory regulations are way down on the to-do list. The current hyper-partisan environment is not likely fertile ground for consensus around these issues.

While we wait to see if there is future action, I think we would all do ourselves a favor to anticipate what future regulations might look like and require of us. I, for one, will place an emphasis on *a priori* establishment of performance characteristics and intended use. Why? Because we may have to show our work to a broader audience at some future point. Validation reports should include better justification for performance characteristics, clinical utility, reference intervals, the intended patient population, and plans for changing and adapting the method as medical knowledge evolves. In this latest effort, the FDA has made it clear what future expectations might be. There is low-hanging fruit that we can incorporate in our method development without embracing all the crazy, expensive requirements that the final rule included.

**Genzen:** This is a key question that many of us are asking: What comes next, and how can we as the laboratory community best prepare for it? Personally, I am hoping that we have an opportunity to have a substantive discussion about what a CLIA-centric LDT regulatory reform proposal could look like. I know some in the clinical laboratory space are nervous about CLIA reform, presumably because it might open a Pandora’s box of potential changes that could disrupt existing frameworks that, for the most part, work remarkably well. I do not think that concern should limit us, however, from being proactive in thinking about how CLIA could be enhanced and modernized to address some of the concerns shared by proponents of LDT oversight by the FDA.

How could we begin that dialogue? I think it is reasonable to start by considering general classes of criticisms coming from some outside the clinical laboratory community. For example, why could CLIA not be updated to specifically address clinical validity requirements? Clinical validity is already addressed in CLIA-centric regulatory frameworks such as the College of American Pathologists (CAP) Laboratory Accreditation Program (LAP) COM.40640 checklist item and the New York State Department of Health Clinical Laboratory Evaluation Program (NYSDOH CLEP) [8–9].

I also see no reason why CLIA regulations could not be similarly updated to address work that many clinical laboratories are already doing with our LDTs. I have previously noted that many clinical laboratories are already required to maintain a list of LDTs per accreditation requirements (e.g., CAP COM.40830), NYSDOH CLEP maintains a public database of NY-approved LDTs, and even the CMS CLIA-application form (CMS form 116) requires laboratories to list the manufacturer of individual assays [10–13]. So, when people say that we have no idea what LDTs are out there, it is important to emphasize that much of this information exists; it is just not collected, collated, or made available in a way that would be easy for the public to review. But this situation could easily be rectified by CMS (or even accreditation agencies) to create greater visibility of what LDTs are being offered and where, and it could even include key performance characteristics for those assays. We do not need FDA oversight of LDTs to address concerns about transparency, but we do need to have an open discussion about how this could most easily be accomplished under CLIA. Now is the time to foster that conversation, and it will require a collaborative, cross-community/cross-industry dialogue that supports novel frameworks. I am ready for that discussion, and I hope others are as well.

**Van Wijk:** We may see a renewed version of the VALID Act introduced in Congress. Alternatively, Congress could choose to modernize CLIA, explicitly codifying that LDTs fall under CMS/CLIA rather than the Federal Food, Drug, and Cosmetic Act. In the meantime, it is important for laboratories to stay informed about policy developments, remain actively engaged with professional organizations such as the ADLM, AMP, and CAP, and continue to advocate for innovation and patient access to LDTs.

**Willrich:** We have seen several regulatory proposals over the years, from the VALID Act to the recent FDA rule to classify LDTs as medical devices in a phased approach, recently vacated by a federal court. LDT regulation remains an unsettled issue. A revised VALID Act could be brought forward, or CLIA could be updated. To prepare, laboratorians should focus on developing robust LDTs with comprehensive documentation. Clinical laboratories should establish or strengthen regulatory affairs to review test development, lab compliance with policies and procedures, and ensure readiness to oversight changes.

***Did your lab take any steps to comply with the FDA final rule while it was in effect? Did you find anything of value in your preparation that you plan to implement, even with the final rule no longer in place?***

**Dietzen:** The litigation of the final rule played out as I oversaw two tertiary-care pediatric institutions with a broad menu of LDTs. Given the evolving political landscape, the lack of answers to vague parts of the final rule, and less-than-concrete reporting/labeling requirements, both of these labs adopted a sort of wait-and-see approach. Laboratorians are trained to have a healthy respect for rules, but there did not appear to be any. At the end of the day, this approach paid off. We did not waste a lot of time in redesigning our method development or quality systems. I am certain that we were not the only ones that played this same hand of poker with the FDA.

**Genzen:** There is always value in learning from experiences and new regulatory proposals and/or requirements. The FDA LDT final rule prompted clinical laboratories to review and evaluate the totality of their LDT menu. Many organizations found value in the lessons learned from those reviews, be they operational, financial, or clinical. Consideration of how an existing quality management system (QMS) may or may not align with FDA quality system requirements (QSRs) was also a valuable experience for many laboratories, although I think it also highlighted how poorly aligned FDA requirements are for most clinical laboratory settings. Clinical laboratories deserve regulatory oversight that complements (rather than hinders) routine operational activities, and that is what I am hoping we achieve going forward.

**Van Wijk:** I kept myself up to date and compiled a list of requirements for compliance, particularly for Phases 1 and 2 (Phase 1: medical device reporting, corrections and removals reporting, and complaint files requirements; Phase 2: registration and listing, labeling, and investigational use requirements) [5], as the institution worked through a standardized approach. One nuance I had previously failed to fully appreciate is the issue surrounding Research Use Only (RUO) components and kits. Per my understanding of the FDA final rule, a clinical laboratory can incorporate one or more RUO components into its LDT, provided those components are appropriately qualified. However, an RUO kit used on its own (i.e., not as a component) and without any modifications may be viewed by the FDA as a “misbranded” in vitro diagnostic product (see 2013 Guidance: Distribution of In Vitro Diagnostic Products Labeled for Research Use Only or Investigational Use Only [14]). Warning letters have been issued by the FDA to manufacturers in such cases [15]. How this is viewed under CLIA is less clear to me, but it is one issue I am now more cognizant of.

**Willrich:** Yes, we invested significant time assessing our processes, cataloging LDTs, and considering if there would be an FDA-approved test. In some cases, we found alternatives that were FDA approved, but they were less optimal—less amenable to workflows, had incomplete analytical coverage when considering a panel or reflex tests, higher degree of interferences, or higher cost. We documented these decisions carefully, and this experience led us to formalize the method selection and validation rationale more explicitly in our assay development plans. Our validations were already rigorous, but the exercise sharpened our focus on traceable, transparent decision-making.

***What standards or guidelines do you follow in your lab when implementing an LDT (e.g., minimum CLIA requirements, Clinical Laboratory Standards Institute (CLSI) guidelines, etc.)?***

**Dietzen:** It is impossible to build sophisticated LDTs with one-size-fits-all rules. Implementing LDTs cannot be broken down to a simple formula. Next-generation sequencing is different than flow cytometry, is different than mass spectrometry. CLIA regulations embody the core principles of analytic validation. These principles include assessment of accuracy, assessment of repeatability, suitability of sample types, intended patient populations, and provision of interpretive criteria. Guidance from CLIA, CLSI, CAP, and International Organization for Standardization (ISO) are all helpful, but the magic sauce comes from highly trained MD and PhD clinical laboratory scientists. LDTs are necessary to fill gaps where no FDA-approved applications exist or where FDA-approved applications are insufficient for their intended clinical purpose. Laboratory professionals with a deep understanding of pathophysiology must custom build these applications. Exceedingly rare conditions require carefully planned validation studies, often in collaboration with other laboratories. Does every component of a quantitative multi-analyte profile require the same imprecision profile? Maybe or maybe not, but a CLSI document or a CAP guideline is not going to fix such problems by themselves. Guidelines are important. Highly trained professionals are more so.

**Genzen:** CLIA performance standards (§493.1253) are an absolute requirement for all US clinical laboratories performing testing on human clinical specimens, and part 2 of this standard, “establishment of performance specifications,” contains the relevant requirements for LDTs [16]. Our laboratory is CAP accredited, and we also have a New York clinical laboratory permit, so we follow LDT requirements associated with both the CAP LAP and NYSDOH CLEP. I have overseen body fluid testing throughout my career (a common type of LDT), and I am a big proponent of the CAP COM.40620 body fluid analysis checklist requirements [17]. I would go so far as to say that some of these concepts should also be incorporated into CLIA regulations directly, particularly around what laboratories can do if one can “reasonably exclude” the existence of matrix interferences [17].

I also note that NYSDOH CLEP LDT requirements include pre-market review [9]. I think this is a perfect illustration of how activities can (and often currently do) occur under CLIA without an additional regulatory layer from the FDA. We also use a number of other clinical laboratory standards regarding test validations, including some from CLSI, as well as best practices observed in the peer-reviewed literature and information presented at scientific conferences. CLSI documents relevant to certain LDTs are outlined on the CLSI website [18]. Speaking of scientific literature: another key point is not just what standards and guidelines we follow as a clinical laboratory, but also our commitment to share what is learned by publishing findings in the peer-reviewed literature. We have published many LDT validations, and there are tangible, practical benefits to having a robust literature to refer to on LDTs using multiple platforms and fluid types (i.e., COM.40620 requirements for reference intervals and exclusion of matrix interferences).

**Van Wijk:** At a minimum, we follow CAP and CLIA requirements; however, additional testing may be required depending on the type of LDT being validated. If relevant CLSI guidelines exist, we refer to those as well.

**Willrich:** We maintain a robust suite of internal validation templates developed by a group of quality specialists, quality coordinators, and developers as roadmaps to LDT validation. The templates adhere to CLIA, CAP checklists, and CLSI guidelines. For LDTs, laboratories essentially act as a test manufacturer and rigorously validate assay performance characteristics. We follow the acronym PARRAsAs [precision, accuracy, reference interval, reportable range, analytical specificity (limit of detection), and analytical sensitivity (interference testing)] as default experiments, as required by CLIA. CAP standards compel laboratories to support any clinical claims of an LDT with data or literature, and that is added to our LDT validation templates. It often takes our laboratories about a year from design plan to test implementation.

***Do you have an existing comprehensive quality management system (e.g., ISO15189)? If yes, please describe your QMS and how it helps ensure quality in your laboratory. If no, please describe barriers preventing implementation in your laboratory.***

**Dietzen:** I think all laboratories strive to establish adequate quality management systems (QMSs). My present and former laboratories take this quite seriously. We adhere to CAP standards. We have robust quality control and proficiency testing standards. We seek constant clinician feedback. We scrutinize all changes in reagent lots and the consequences of instrument maintenance. LDTs demand this kind of oversight. Today, I think we are too often judged by the content of our documents and not by execution of the program described by the document. I think CLIA is a minimal set of standards. To build the most sophisticated LDTs, subscription to a higher level of standards is necessary. The barriers here are not access to a set of QMS regulations, but rather adequate implementation experience. People, lab directors, and clinical laboratory scientists make the documents dance.

**Genzen:** Yes, our laboratory has ISO 15189 accreditation [19]. Concepts in ISO 15189 reflect some (but not all) of the QSRs that would have been applied to clinical laboratories in stage 3 of the FDA LDT final rule. In that respect, laboratories with ISO 15189 accreditation are already familiar with concepts such as corrective and preventative actions and supplier controls, as two key examples. Additional layers of accreditation requirements, however, can increase overall operational costs, which is why I also support concepts of streamlining best practices within existing laboratory accreditation programs.

**Van Wijk:** While we are not ISO 15189 accredited, we are accredited by CAP and follow all standards and checklist requirements. This means we have a documented organizational structure with defined roles and responsibilities, perform regular self-inspections, ensure personnel training and competency, and maintain a comprehensive document control and record retention system. We maintain procedures and documentation for all aspects of the testing that we perform, such as standard operating procedures, verification and validation of new tests, ongoing quality control and proficiency testing, instrument-to-instrument comparisons, instrument maintenance, and new lot and calibration verification. In addition, we investigate incident reports, perform root-cause analyses, monitor quality indicators, and carry out corrective actions and quality improvement projects. Note that these are representative examples and not an exhaustive list. Therefore, I believe that compliance with CLIA, and especially CAP standards, inherently requires a robust quality system—one that we in the clinical laboratory community often do not explicitly label as such.

**Willrich:** Yes, we operate as an academic medical center with a reference laboratory, which performs high-complexity testing in multiple subspeciality laboratories under a comprehensive quality management system. Our QMS is a virtual collection of quality policies, procedures, and processes organized by Quality System Essentials (QSEs) spanning structure, process, and outcome. More than 60 individual laboratories within our department follow these global documents and apply them to their practice and workflows, supplemented by division-specific procedures where appropriate. In a high-volume environment, there are always questions on how to improve processes, if what we are doing to comply with a certain checklist is sufficient; having a QMS, along with quality management coordination personnel, is essential to uphold high quality standards in the laboratory and drive continuous improvements. Developing an LDT is a first step. Maintaining its quality and relevance over time is also critical. Proficiency testing is not available for several LDTs, so planning performance of alternative assessments or sample exchange with other laboratories before test implementation and throughout the test life cycle is an important part of the QMS.

***Does your laboratory assess clinical validity of new LDTs? If so, how do you assess this? Do you think clinical validity assessment is necessary for all LDTs? For select LDTs? Not necessary?***

**Dietzen:** This is a tricky point, and one that the FDA enjoyed using to undermine the quality of LDTs in use today. I think there are LDTs today that are not clinically relevant. Many of these are deployed in the direct-to-consumer market. The FDA and the Federal Trade Commission (FTC) should absolutely work to remove these from the market. In my experience, the LDTs that I build are based upon clinical demand and have clear clinical validity. For example, pediatric laboratories all need the capability to assess phenylalanine concentrations in Phenylketonuria (PKU) to prevent cognitive damage to affected patients. We have mountains of evidence supporting the control of blood phenylalanine as a tool to keep these children healthy. Would the FDA demand we do this again? Would we require a large, randomized, double-blind controlled trial of treating children with PKU with or without phenylalanine measurement to prove clinical validity? Such a trial would be unethical at the least. In some cases, however, clinical relevance is not that easy to ascertain. In some cases, LDTs are not diagnostic. They may provide small clues and build on other clinical and laboratory data to help reach very difficult or rare diagnoses. Are these any less clinically valid? There are many ways to evaluate and show clinical validity. Working with clinicians to build useful tools is the best way to ensure it.

**Genzen:** As noted above, assessment of clinical validity is already a requirement of both the CAP LAP and the NYSDOH CLEP. However, I think it is very important that future regulatory structures allow flexibility in how clinical validity is demonstrated. For example, use of peer-reviewed literature—as opposed to generating new clinical validity data every time an assay is developed—should be adequate if clinical validity has already been demonstrated for that analyte elsewhere. Most clinical laboratories will not have the financial resources to support full clinical trials. Assessment of clinical validity already works well in a pre-market model under NYSDOH CLEP with the associated risk attestation form, so I would suggest this as a model for how other CLIA-oversight proposals could address clinical validity as well [20].

**Van Wijk:** We generally perform LDTs for analytes that are clinically well-established. I believe that clinical validity assessment is necessary for all LDTs and that this requirement can often be satisfied for existing analytes through evidence found in peer-reviewed medical literature and clinical guidelines. Similar to instrument manufacturers, clinical laboratories should be able to support their clinical claims with robust evidence. We want to avoid a situation in which the clinical relevance of measuring an analyte is questionable, especially in the direct-to-consumer setting. While the FDA’s final rule clearly stated that direct-to-consumer tests were never subject to LDT enforcement discretion, their current regulatory oversight status is unclear to me when offered as a *service* within a single CLIA-certified laboratory.

**Willrich:** CLIA requires robust analytical method validation but does not guarantee clinical validity. Other guidelines such as CAP standards suggest that clinical claims of LDTs be supported by data or previous publications. We assess clinical validity in most cases, although the depth and methods vary. Strategies include literature review, comparison with established tests, and review of medical records and clinical history; more recently, we have started developing AI tools that can streamline chart review to assist with this task.

Whether clinical validity assessment is necessary depends on the context. For example, the clinical relevance of monoclonal proteins as tumor markers in multiple myeloma is well-established; thus, when implementing MASS-FIX, our focus was on analytical validation rather than proving clinical validity again.

Developing robust clinical validity data for novel biomarkers can be extraordinarily resource intensive. For example, when exploring Cerebrospinal fluid (CSF) kappa free light chains as an alternative to oligoclonal banding in multiple sclerosis, it took four years, thousands of samples, and a large multidisciplinary team to establish comparable diagnostic performance. Such efforts are feasible only at a handful of centers and require substantial investment.

***Do you think the current regulatory framework for LDTs is sufficient to ensure the tests available in the market are safe and effective? If yes, why? If no, what do you think is missing and what changes do you recommend?***

**Dietzen:** I think that the current CLIA framework supplemented with CAP and/or ISO standards is adequate for well-trained, conscientious professionals to build safe, effective LDTs. I think the vast majority of LDTs are built this way. The FDA cited several examples of LDTs that they deemed defective in some way, but much of this was disinformation [21]. Calling out the inaccuracy of prenatal cell-free DNA screening tools failed to mention the fact that these are screening tests designed to optimize sensitivity over specificity. The bulk of my frustration with the FDA guidance was trying to figure out what problem the FDA was trying to solve. Most LDT development is not done to skirt the FDA. It is done to fill holes that FDA-approved methods fail to address. That said, there are unscrupulous players that use the cover of the “LDT pathway” to avoid appropriate regulatory scrutiny. These limitations can be addressed by requiring a bit more documentation of clinical validity and better evidence that LDT builders possess the necessary expertise to build and monitor very sophisticated analytic systems. The best remedy is to do this through the existing CLIA regulatory structure, if it still exists in the next few years.

**Genzen:** I believe that the current regulatory framework has been sufficient to ensure the safety and effectiveness of LDTs, but there is always room for improvement and to provide confidence for the public in ensuring health and wellness with diagnostic testing. The public deserves transparency, so I am in favor of finding the right mechanism to achieve that if the information and processes are not too burdensome for laboratories to comply with such requirements. FDA-centric product labeling seemed excessive and not quite the right model—the federal court picked up on this as well—but creating greater transparency for key performance characteristics and test information seems reasonable and in the broader public interest. But again, I think this is easily accomplishable under CLIA and at far lower cost if regulators and legislators are amenable to this type of reform. We, as a community, should continue to advocate for our patients’ diagnostic needs.

**Van Wijk:** I believe CLIA should be modernized to include a minimum standard for clinical validity assessment. Additionally, it should incorporate a specific experience requirement for laboratory staff involved in the development of LDTs, as well as for laboratory directors responsible for overseeing their implementation and performance. This is particularly important for LDTs that utilize specialized technologies such as next-generation sequencing, mass spectrometry, and flow cytometry.

**Willrich:** The framework is broad and open to various interpretations. Most clinical laboratories are highly diligent in validating and documenting their tests, and the vast majority of tests have outstanding benefit to patients, to vulnerable populations, and to improved diagnosis of rare disorders. That said, the system already imposes high costs on institutions and remains opaque to the public. As a patient or non-expert, it is difficult to appreciate the rigor that goes into LDT validation. Patient safety and test reliability are top of my mind for LDT regulation. My career in laboratory medicine has focused on the development and implementation of LDTs, not necessarily on broader policy development for their regulation. Finding a one-size-fits-all approach for LDT regulation seems difficult and impractical—something that would require effort from multiple minds with different backgrounds and significant legal, political, and regulatory background. Perhaps making validation data available for peer review could incentivize greater transparency and standardization. Many colleagues advocate for modernizing CLIA rather than expanding FDA authority, and others have proposed adding clinical validity data under an updated CLIA regulation. This could strengthen LDT oversight while reducing ambiguity and could strike a balance between ensuring safety and preserving innovation.

## Conclusion

As highlighted by the panel of experts interviewed for this article, LDTs play a critical role in patient care because commercial IVDs do not meet all clinical testing needs. Gaps include areas such as toxicology, proteomics, inborn errors of metabolism, and pediatric testing, among others. The experts agreed that the increased oversight proposed by the FDA in the now-vacated final rule would have curtailed innovation, limited test availability, and increased costs for laboratories, ultimately harming patients. Although LDTs are generally safe and effective under the existing framework, the panel noted that there is room for greater transparency and improved documentation of clinical validity. Most laboratories follow rigorous internal validation protocols that align with professional practice guidelines, maintain robust quality management systems, and often exceed minimum regulatory requirements. Overall, the expert consensus is that any regulatory reform should enhance oversight and transparency without imposing excessive burdens on clinical laboratories and should occur within the existing regulatory framework under CLIA, with input from expert clinical laboratory professionals.

Given the importance of LDTs and the persistent push for regulatory reform over the past decade, the matter of increased oversight is far from settled. As laboratorians, we should monitor this issue closely and continue to apply the latest professional practice guidelines when designing and validating LDTs. We should also work constructively with Congress and regulatory agencies, advocating for a balanced approach that ensures appropriate oversight without further straining laboratories’ already limited resources.

## Data Availability

This article is an opinion piece and did not involve data collection, data analysis, or custom code.

## Abbreviations

CLIA: Clinical Laboratory Improvement Amendments
CMS: Centers for Medicare & Medicaid Services
FDA: US Food and Drug Administration
IVCT: In vitro clinical tests
IVD: In vitro diagnostic
LDT: Laboratory-developed test
VALID Act: Verifying Accurate Leading-Edge IVCT Development Act
